# Exome Sequencing in Classic Hairy Cell Leukaemia Reveals Widespread Variation in Acquired Somatic Mutations between Individual Tumours Apart from the Signature *BRAF* V(600)E Lesion

**DOI:** 10.1371/journal.pone.0149162

**Published:** 2016-02-12

**Authors:** Nicola J. Weston-Bell, Will Tapper, Jane Gibson, Dean Bryant, Yurany Moreno, Melford John, Sarah Ennis, Hanneke C. Kluin-Nelemans, Andrew R. Collins, Surinder S. Sahota

**Affiliations:** 1 Tumour Immunogenetics Group, Cancer Sciences Unit, Faculty of Medicine, University of Southampton, Southampton, United Kingdom; 2 Genetic Epidemiology and Genomic Informatics Group, Human Genetics, Faculty of Medicine, University of Southampton, Southampton, United Kingdom; 3 Centre for Biological Sciences, Faculty of Natural and Environmental Studies, University of Southampton, Southampton, United Kingdom; 4 Department of Preclinical Sciences, Faculty of Medical Sciences, University of The West Indies, St. Augustine, Trinidad and Tobago; 5 Department of Hematology, University Medical Center Groningen, University of Groningen, Groningen, The Netherlands; Cornell University, UNITED STATES

## Abstract

In classic Hairy cell leukaemia (HCLc), a single case has thus far been interrogated by whole exome sequencing (WES) in a treatment naive patient, in which *BRAF* V(600)E was identified as an acquired somatic mutation and confirmed as occurring near-universally in this form of disease by conventional PCR-based cohort screens. It left open however the question whether other genome-wide mutations may also commonly occur at high frequency in presentation HCLc disease. To address this, we have carried out WES of 5 such typical HCLc cases, using highly purified splenic tumour cells paired with autologous T cells for germline. Apart from *BRAF* V(600)E, no other recurrent somatic mutation was identified in these HCLc exomes, thereby excluding additional acquired mutations as also prevalent at a near-universal frequency in this form of the disease. These data then place mutant BRAF at the centre of the neoplastic drive in HCLc. A comparison of our exome data with emerging genetic findings in HCL indicates that additional somatic mutations may however occur recurrently in smaller subsets of disease. As mutant BRAF alone is insufficient to drive malignant transformation in other histological cancers, it suggests that individual tumours utilise largely differing patterns of genetic somatic mutations to coalesce with *BRAF* V(600)E to drive pathogenesis of malignant HCLc disease.

## Introduction

Hairy cell leukaemia (HCL) is a rare B-cell neoplasia with a distinctive morphology, displaying extensive cytoplasmic projections that give the disease its name [1–2]. It presents in the main as a typical or classic form of disease (HCLc) that can be distinguished from the atypical or variant HCL subtype (HCLv) by the specific immunophenotypic markers CD25, CD123, and ANXA-1. The HCLv subtype accounts for ~10% of all HCL disease cases, and differs in disease behaviour, associating more frequently with refractory response to therapy [1–3]. It is as yet not fully known how these differing HCL disease forms arise, and whether the precise molecular and cellular pathways that drive the pathogenesis of HCLc and HCLv disease subtypes are related or vary markedly.

In early work on HCL origins, we as well as others probed this question by focusing on analysis of immunoglobulin (Ig) heavy chain variable region (*IGHV*) genes. Ig variable domains are crucial in recognising antigen via the B-cell receptor (BCR), and these domains are specifically encoded by rearranged *IGHV* genes paired with rearranged light chain variable region (*IGVL*) genes [4]. The germline repertoire of *IGHV* genes alone comprises several families based on shared homology (*IGHV1* to *IGHV7*), and each family comprises varying numbers of gene segments which are referred to in notation depending on locus distribution (e.g. gene segment 34 as *IGHV4-34* in family *IGHV4*) [5]. Across HCL disease, where tumours are invariably monoclonal, the repertoire of tumour-derived *IGHV* gene use is varied and compared with the normal B-cell repertoire, use of *IGHV3-21*, *IGHV3-30* and *IGHV3-33* appears elevated suggesting possible selection pressure of antigen on the cell of origin in this malignancy [6]. Regarding *IGHV* gene usage, ~10% of HCLc cases utilise the *IGHV4-34* gene (hereafter referred to as HCLc-*IGHV4-34* cases), and importantly use of the *IGHV4-34* gene segment specifically associates with a poorer outcome of disease and suboptimal response to therapy [7]. These insights from *IGHV* gene usage implicate distinctive pathways in clonal origins in HCL.

The nature of oncogenic events that drive malignant transformation in HCL had until recently received scant genome-wide attention. Seminal insights have emerged first in HCLc disease. In this subset, a mutation in *BRAF* V(600)E was identified in whole exome sequencing (WES) of a single typical case at disease presentation [8], and due to the known prominence of this mutation more broadly in cancer, led to its selection and verification by conventional PCR-based screens in a larger number of HCLc tumours to establish it as a signature genetic mutation in this disease subtype [8,9,10]. Although these studies confirmed the occurrence of *BRAF* V(600)E as near universal in HCLc disease, interestingly and in marked contrast, in HCLv and in the unusual HCLc-*IGHV4-34* cases, *BRAF* V(600)E was found to be invariably absent suggesting segregated pathways in the origins of these different forms of HCL disease [11,12]. It left open however the question of the nature of driver mutation(s) in the atypical HCL subtypes.

More recently this question has been resolved to a large extent. Waterfall *et al* carried out WES of 10 HCLv and HCLc-*IGHV4-34* cases, and in these *MAP2K1* mutations emerged as a common somatic variant (SV) [13]. By examining *MAP2K1* mutations more broadly by standard Sanger protocols, the overall frequency of mutations in *MAP2K1* was identified as 48% in a panel of HCLv/HCLc-*IGHV4-34* tumours (n = 21) [13]. *MAP2K1* mutations were however rare in typical HCLc that lacked the *IGHV4-34* gene, to separate disease subsets based on the acquisition of distinctive somatic mutations [13]. The *MAP2K1* mutation yielding a p.Cys121Ser coding shift was the most common in the combined HCLv/HCLc-IGHV4-34 tumour panel, and *MAP2K1* mutations associated with predicted constitutive enzymatic activity [13]. Apart from *MAP2K1*, other gene mutations were far less common in HCLv/HCLc-*IGHV4-34* exomes [13], indicating that the *MAP2K1* mutation is dominant and a driver in atypical HCL origins and progression (i.e. in HCLv/HCLc-*IGHV4-34* tumours).

These recurrent gene mutations in classic and atypical HCL disease highlight specific cellular signalling conduits in disease origins. In its wild type form, the *BRAF* gene encodes the BRAF serine/threonine kinase which phopsphorylates and activates its substrates MEK1/2, and thereby transduces signals via the RAS-RAF-MEK-ERK mitogen-activated protein kinase (MAPK) pathway [14]. In this signalling pathway, MEK1 (also known as MAPKK1, mitogen-activated protein kinase kinase 1) is encoded by the *MAP2K1* gene [15]. *BRAF* and *MAP2K1* genes have emerged as commonly mutated in HCLc or atypical HCL respectively.

In attempting to correlate exome findings in HCLc with atypical disease further however, a marked limitation emerged, as thus far only a single exome has been analysed in presentation HCLc disease [8]. This left open the question whether other mutation(s) might also occur across the genome that are recurrent, in addition to *BRAF* V(600)E. Additional common driver mutations in typical HCLc tumours appeared highly probable, given the findings that although the *BRAF* V(600)E mutation is an early founder event in both benign melanocytic naevi and pre-malignant colonic polyps, it is nevertheless insufficient for full malignant transformation in these tumours [16]. These considerations suggested a possibility that additional driver(s) events may be required to synergise with *BRAF* V(600)E to progress malignant transformation in typical HCLc, that would require identification at a genome-wide level. To address this, we have carried out WES in 5 typical HCLc cases, in each comparing tumour exome with matched germline.

## Materials and Methods

### Patient Samples

#### Ethics

Ethical approval for the study was obtained from institutional bodies. The HCLc samples were obtained by Professor H. Kluin-Nelemans in the Department of Hematology, University Medical Center Groningen, Netherlands, and provided for study use approved under Ethical Review by the National Research Ethics Service Committee South Central (Southampton A) Reference M228/02/t (Amendment 7). The HCLc samples comprise an existing holding collected in 1980–1990 and for which consent is waived by the Human Tissue Authority. The University of Southampton UK holds Human Tissue Authority licence 12009.

#### Samples

Typical HCL disease was diagnosed by clinical criteria, bone marrow and spleen histology, neoplastic cell morphology, cytochemical analysis and immunophenotype [17] (and Table 1). Following splenectomy, splenic lymphocytes were disaggregated and stored in liquid N_2_ until use. After thawing, samples were resuspended in complete RPMI medium (cRPMI) (RPMI 1640, 20% foetal calf serum, 1 mM sodium pyruvate MEM, 0.1 mM MEM non-essential amino acids, 2 mM L-glutamine (Gibco Invitrogen, Paisley, UK)) and allowed to stabilise at 37°C in 5% CO_2_ for at least 1 h prior to use. Viability was assessed by trypan blue exclusion.

**Table 1:** Tumour characteristics of classic Hairy cell leukaemia cases analysed by whole exome sequencing. ND: not done. Surface immunophenotype, *IGHV* gene use and % homology to germline was determined as reported [17,18,19].

Tumour	Surface immunophenotype							% tumour	sIg expression	*IGHV*	% Homology to germline	*BRAF* V(600)E^#^ Sanger	Purity post-sort	
	CD19	CD11c	CD103	CD27	CD123	CD25	ANXA1						HCLc	T
3T	+	+	+	-	ND	+	ND	83	G^+++^M^++^κ^+++^	3–30	96.10%	+	92.90%	98.60%
5T	+	+	+	-	ND	ND	ND	80	A^+++^G^+++^M^+^κ^++^λ^+++^	3–15	92.20%	+	98.20%	96.80%
9T	+	+	+	-	ND	ND	ND	65	A^+++^D^+++^G^+++^M^+++^κ^+++^	3–30	92.70%	+	97.80%	96.70%
S4	+	+	+	-	+	+	+	71	D^+++^λ^++^	3–30	96.90%	+	98.30%	99.2%
S6	+	+	+	-	+	+	+	65	D^+++^λ^++^	3–7	94.10%	+	97.80%	99.0%

^#^*BRAF* V(600)E mutation was first confirmed in each case by conventional Sanger sequencing as we have described previously [18, 19].

### Immunophenotyping

Multiparameter flow cytometric immunophenotype was performed as described previously [18], with sIg expression analysed on CD19^+^CD11c^HI^ or CD19^+^CD11c^HI^CD103^+^ HCL cells (Table 1). Additional markers were assessed using CD25-PE (BD Biosciences, Oxford, UK), CD27-PE (BD) and CD123-PE (BD) antibodies on CD19^+^CD11c^HI^CD103^+^ cells (Table 1). Data were analysed using FACSDiva (BD) and FlowJo (Tree Star Inc., Ashland, OR, USA). ANXA1 detection was performed as previously described [19]. Briefly, ANXA1 was detected either by western blot or immunocytochemistry using the antibody ‘Purified mouse anti-Annexin I’, clone 29/Annexin I (BD Transduction Laboratories, Oxford, UK) (Table 1).

### *IGHV* analysis

*IGHV* sequencing was performed as reported previously [20], using IMGT V-QUEST (http://www.imgt.org/IMGT_vquest/vquest?livret=0&Option=humanIg) for *IGHV* donor germline gene derivation (Table 1). None of the HCLc cases utilised *IGHV4-34* germline *IGHV* gene, and all tumours uniformly exhibited somatically hypermutated *IGHV* rearranged genes.

### FACS purification of HCL tumour cells and germline T cells

**Fig 1:**
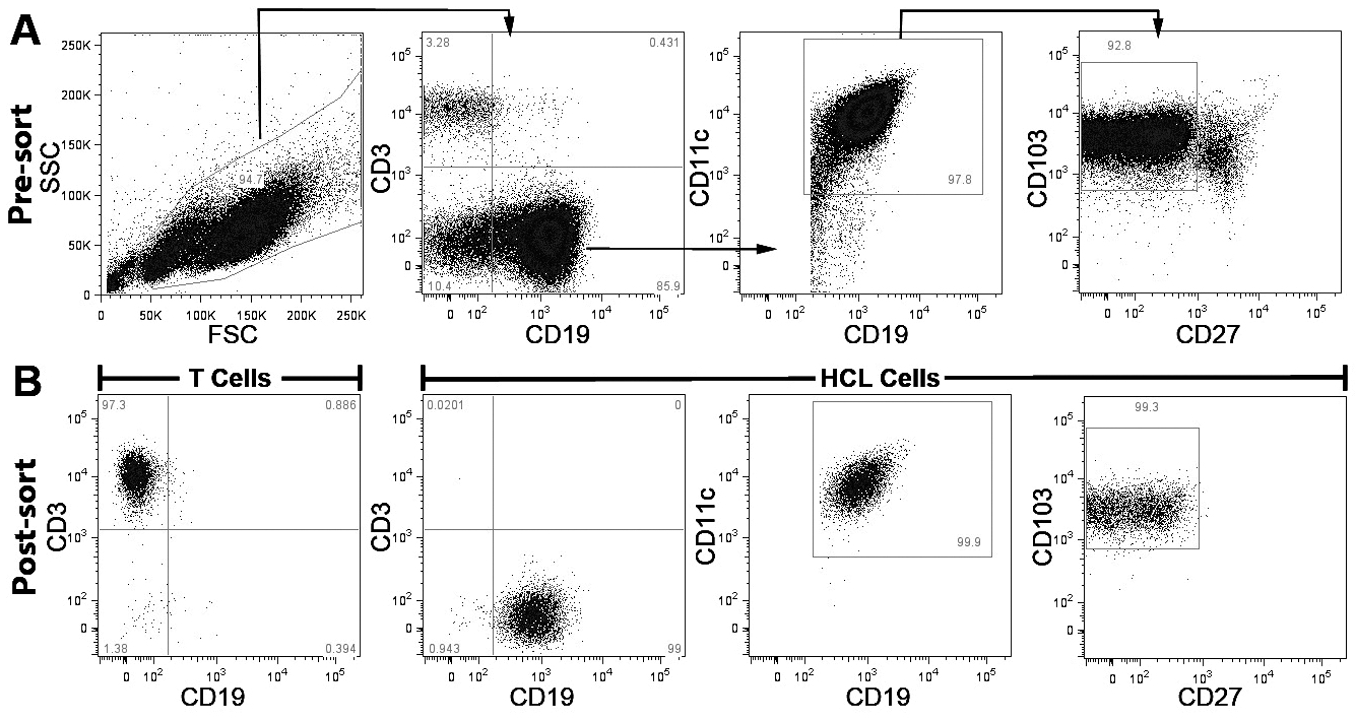
Gating strategy in flow cytometry to purify HCLc tumour cells and matched germline T cells. Splenocytes were stained with anti-CD19-eFluor450, anti-CD3-PE, anti-CD11c-APC, anti-CD27-APC-eFluor780 and anti-CD103-FITC prior to sorting on the FACSAria. The plots show sorting on a representative HCLc case. **A:** displays pre-sorted mononuclear cells, with CD19^+^CD11c^+^CD103^+^CD27^-^ HCLc tumour cells 78.0% of sample and CD3+ T cells 3.3%. Cells were initially gated on FSC/SSC (**A**, left) then either CD3+ for T cells or CD19+ for B cells (**A**, second left). CD19+ cells were subsequently gated as CD11c+ (**A**, second right), then CD103+ and CD27- (**A**, right) to capture high purity HCL cells. In **B** post-sorted cells were checked for purity: T cells were purified at 97.3% (left panel) and HCLc cells purified at 98.2% (right, three panels). Data is representative of cell fraction isolation in each of 5 samples.

HCL samples were stained for CD19-eFluor450 (eBioscience, Hatfield, UK)/CD19-V450 (BD), CD11c-APC (BioLegend, London, UK), CD103-FITC (BioLegend/BD), CD27-APC-eFluor780 (eBioscience) and CD3-PE (BD/eBioscience) as described for immunophenotyping. HCLc tumour cells were sorted and selected as a CD19^+^CD11c^HI^CD103^+^CD27^-^ population and germline T cells from the same sample as CD3^+^ using the FACS Aria I (BD) (Fig 1). Sorted populations were re-analysed for purity on the FACS Aria I: purity of HCLc sorts was 92.9–98.3% and of T cells 96.7–100% (Fig 1 is a typical example). Data were analysed using FACS Diva (BD) and FlowJo (TreeStar).

### Genomic DNA extraction

Genomic DNA was immediately extracted from sorted HCLc and T cell populations respectively using the DNeasy Blood and Tissue kit (Qiagen, Crawley, UK), according to the manufacturer’s protocol. Extracted DNA concentration was assessed by spectrophotometry (NanoDrop ND1000, Labtech, Uckfield, UK) and the DNA stored at -20°C until use. HCLc DNA was initially used to verify the *BRAF* V600E mutation by conventional PCR and Sanger sequencing (Table 1) as we have reported previously [19].

### Sanger Sequencing to Verify Somatic Variants

>30% of identified somatic variants (SVs) were selected at random for validation by PCR amplification (primers available on request) and capillary Sanger sequencing (Tables 2 & 3). 50 ng DNA was used in multiplex PCRs with 35 cycles of 95°C for 30 s, 58°C for 30 s, 72°C for 1.5 mins. T cell germline DNA was used as control. PCR products were separated by electrophoresis on a 2% w/v agarose gel and extracted from excised bands using Qiagen gel extraction kit, and subjected to Sanger sequencing using BigDye Terminator v1.1 with the AB3130*xl* Genetic Analyser (both Applied Biosystems, Life Technologies, Paisley, UK). Data were analysed using MacVector (Accelrys, San Diego, CA, USA) and 4Peaks software (Mek&Tosj, Amsterdam, The Netherlands).

**Table 2:** Non-synonymous somatic variants identified in HCLc tumour exomes.

Sample	Total	*Known	*Novel	^#^Unflagged	Validated
3T	19	7	12	10	5
5T	32	6	26	22	4
9T	14	7	7	7	4
S4	34	18	16	10	1
S6	19	12	7	4	2
Total	118	50	68	53	16

*Known and *Novel indicate presence or absence of SV respectively in the following databases: dbSNP135, 1000 genomes, Complete Genomics 46, the NHLBI GO Exome Sequencing Project, and an in-house database of 279 non-cancer exomes.

^#^Unflagged variants are located outside of repetitive DNA and have not been observed at a frequency of ≥10% in any of 61 germline exomes as determined by inspection in the interactive genome viewer.

**Table 3:** Novel nonsynonymous somatic variants identified in HCLc exomes from paired germline analysis. Annotations: COSMIC Catalogue of Somatic Mutations in Cancer; Novel = not present in dbSNP135, 1000 genomes, Complete Genomics 46, the NHLBI GO Exome Sequencing Project), and an in-house database of 279 non-cancer exomes. Functional predictions: PolyPhen-2.

Sample	Gene	Nucleotide	Amino acid	Waterfall et al [13]	COSMIC	Germline mutant reads (%)	Tumour mutant reads (%)	Somatic *p*-value	Functional prediction	Validated
3T	***BRAF***	A>T	**p.V600E**	p.V600E	Y**	5 (2.45)	81 (48.21)	5.37x10^-28^	Possibly deleterious	n
3T	*ZFP36L1*	T>A	p.L17F, p.L86F		X*	3 (1.76)	52 (40.94)	3.27x10^-19^	Possibly deleterious	y
3T	*CREBBP*	G>A	p.P1072L, p.P1110L	p.C1383fs	X*	1 (0.54)	53 (31.36)	1.74x10^-18^	Probably deleterious	y
3T	*DMXL2*	T>C	p.E471G,		X**	5 (3.11)	60 (42.25)	4.18x10^-18^	Neutral	y
3T	*DDX56*	C>T	p.R387H, p.R427H		X*	1 (1.12)	31 (41.89)	6.69x10^-12^	Deleterious	n
3T	^#^*CNTNAP4*	G>A	p.E555K		X*	1 (1.05)	30 (38.46)	1.99x10^-11^	Neutral	y
3T	*GMNC*	G>A	p.S81F		X	1 (0.95)	32 (28.07)	1.40x10^-9^	Deleterious	y
3T	*CNOT10*	G>A	p.E559K, p.E499K		X*	1 (2.38)	22 (62.86)	2.58x10^-9^	Possibly deleterious	n
3T	*DUSP2*	G>A	p.Q274X		X*	1 (1.64)	10 (21.28)	9.66x10^-4^	Probably deleterious	n
3T	*LCA5*	T>A	p.D677V		X*	1 (2.08)	10 (23.26)	2.066x10^-3^	Neutral	n
5T	*STARD4*	T>C	p.T12A		Y	1 (0.33)	115 (42.59)	1.51x10^-43^	Neutral	n
5T	***BRAF***	A>T	**p.V600E**	p.V600E	Y**	3 (1.35)	82 (50.31)	1.47x10^-33^	Possibly deleterious	n
5T	*PEX1*	C>T	p.R1208Q		X**	7 (3.63)	71 (39.23)	6.77x10^-19^	Neutral	n
5T	*FOXA1*	G>T	p.Y207X		X*	2 (1.54)	47 (47.96)	9.23x10^-19^	Probably deleterious	y
5T	*KBTBD7*	C>T	p.R133H		Y	0 (0)	50 (45.45)	3.79x10^-18^	Deleterious	y
5T	*FAT3*	G>C	p.R3306T		X*	0 (0)	36 (52.94)	7.21x10^-15^	Neutral	n
5T	*POLD2*	G>A	p.Q5X, Q40X		X*	1 (0.84)	44 (36.97)	1.18x10^-14^	Probably deleterious	y
5T	*NCKAP1*	T>A	p.I188F, p.I182F		X*	1 (0.40)	33 (14.54)	1.38x10^-10^	Neutral	n
5T	*ABCC1*	C>A	p.S667Y		X*	0 (0)	30 (25.00)	3.47x10^-10^	Neutral	y
5T	*MAP1A*	G>T	p.E108X		X**	0 (0)	26 (13.20)	5.03x10^-9^	Probably deleterious	n
5T	*KIAA1755*	T>C	p.E440G	p.R897H	Y	0 (0)	12 (37.50)	9.11x10^-8^	Neutral	n
5T	*SDHAF2*	C>T	p.P54S		Y	0 (0)	17 (20.00)	1.23x10^-7^	Deleterious	n
5T	*LAPTM5*	C>T	p.C13Y		X*	1 (0.65)	18 (13.85)	4.49x10^-6^	Possibly deleterious	n
5T	*MAGEC3*	T>G	p.I279S		X**	0 (0)	16 (11.59)	8.06x10^-6^	Probably deleterious	n
5T	*CDKN1B*	C>G	p.T198R		Y	0 (0)	15 (19.48)	1.30x10^-5^	Deleterious	n
5T	*ACTB*	G>A	p.H73Y		X*	0 (0)	11 (31.43)	1.96x10^-5^	Possibly deleterious	n
5T	*SLC22A24*	A>G	p.L202P		Y	0 (0)	15 (10.42)	3.03x10^-5^		n
5T	*SPATA5L1*	G>A	p.D6N,		Y	1 (2.94)	23 (38.98)	4.70x10^-5^	Neutral	n
5T	*MAPK15*	G>A	p.G351D		X*	0 (0)	10 (30.30)	8.44x10^-5^	Probably deleterious	n
5T	*TMSB4X*	G>A	p.A8T		Y	0 (0)	11 (13.58)	3.17x10^-4^	Neutral	n
5T	*FAM108B1*	T>A	p.Y130F		X*	1 (1.33)	13 (15.12)	1.385x10^-3^	Deleterious	n
5T	*TAF3*	G>C	p.D467H		X*	0 (0)	9 (15.00)	1.939x10^-3^	Deleterious	n
9T	*SLC22A8*	T>G	p.T353P, p.T444P, p.T321P		Y	0 (0)	63 (48.84)	4.63x10^-25^	Deleterious	y
9T	***BRAF***	A>T	**p.V600E**	p.V600E	Y**	6 (3.39)	78 (44.32)	1.15x10^-21^	Possibly deleterious	n
9T	*OGFOD1*	C>A	p.P495T		Y	1 (0.74)	59 (44.36)	7.56x10^-21^	Deleterious	y
9T	*ATP1A4*	C>G	p.R431G		X*	0 (0)	29 (30.21)	4.16x10^-10^	Deleterious	y
9T	*GRHL1*	A>G	p.S95G		Y	1 (1.61)	30 (41.67)	4.25x10^-9^	Neutral	n
9T	*C17orf80*	C>T	p.H257Y		Y	0 (0)	27 (38.03)	2.61x10^-8^	Deleterious	y
9T	*RASL12*	C>A	p.E54X		Y	0 (0)	19 (13.57)	6.54x10^-7^	Probably deleterious	n
4	*CHD7*	G>A	p.R1189H		Y*	0 (0)	104 (47.49)	4.20x10^-39^	Deleterious	n
S4	*SCD5*	C>T	p.R247H		Y	1 (0.44)	74 (39.57)	1.10x10^-28^	Probably deleterious	n
S4	***BRAF***	A>T	**p.V600E**	p.V600E	Y**	0 (0)	65 (49.24)	3.01x10^-28^	Possibly deleterious	n
S4	*CLEC6A*	A>G	p.H165R		Y	2 (1.13)	55 (47.83)	1.84x10^-24^	Deleterious	n
S4	*DUSP27*	G>A	p.S567N		X*	0 (0)	37 (60.66)	1.82x10^-19^	Neutral	n
S4	*ZFP36*	C>T	p.S34F		X*	0 (0)	25 (31.25)	1.81x10^-10^	Possibly deleterious	y
S4	*ZFYVE21*	G>A	p.V165M		Y	0 (0)	25 (44.64)	2.14x10^-10^	Neutral	n
S4	*NUDT6*	A>C	p.V160G		Y	0 (0)	21 (24.71)	1.32x10^-9^	Deleterious	n
S4	*TPPP2*	A>T	p.I39F		Y	0 (0)	20 (45.45)	1.25x10^-7^	Possibly deleterious	n
S4	*ISX*	G>A	p.G12D		X*	0 (0)	10 (40.00)	1.68x10^-4^	Neutral	n
S6	***BRAF***	A>T	**p.V600E**	p.V600E	Y**	1 (0.68)	43 (55.84)	9.29x10^-24^	Possibly deleterious	n
S6	*CRYBB1*	G>A	p.P27S		Y	0 (0)	7 (31.82)	1.16x10^-4^	Deleterious	y
S6	*ADIPOR2*	C>T	p.R331W		X*	0 (0)	6 (17.14)	3.452x10^-3^	Deleterious	y
S6	*MUC5B*	C>T	p.P2096L	p.V5587I	X*	0 (0)	4 (10.00)	1.9875x10^-2^	Neutral	n

^#^Gene: listed as a possible false positive [25]. COSMIC: Y = mutation is reported; X = mutation is not present but other somatic mutations are listed in this gene in COSMIC.

*COSMIC: gene contains a mutation that occurs in haematopoietic and lymphoid tissue.

**COSMIC: gene contains a mutation that occurs in hairy cell leukaemia.

### Whole Exome Sequencing

Genomic DNA from HCLc tumour cells and T cells for germline was analysed by WES. Whole exome enrichment was performed with either Agilent SureSelect (50 Mb) (Agilent technologies, CA, USA) or Roche NimbleGen (44.1 Mb) (Roche NimbleGen, WI, USA). The resulting exome libraries were sequenced on the Illumina HiSeq platform with paired-end 100 bp reads to an average depth of 120-134x and with 88–90% of the target bases being covered by 20 or more reads, which is sufficient for confident SV calling (Tables 3 & 4). Sample contamination was estimated to be less than 3% and 0.5% when libraries were prepared using NimbleGen and SureSelect respectively.

**Table 4:** Average exome sequence coverage metrics from paired HCLc exomes.

	Germline	Tumour
Total 100 bp read sequences	111,243,578	101,835,369
Mapped to target	78,593,158	69,852,536
Mapped to target (%)	70.7	68.3
Mapped to target +/-150 bp (%)	90.2	87.4
Mean coverage	133.7	119.5
Target bases with coverage >20 (%)	89.6	87.9

Exome sequence reads were deposited as bam files (accession no. E-MTAB-4313) to the public database ArrayExpress (www.ebi.ac.uk/arrayexpress).

### Bioinformatics

Raw exome sequence data, in the form of FASTQ sequence files, for paired germline and tumour samples were aligned to the reference genome sequence (hg19) using NovoAlignMPI (v3.0). The aligned data were quality controlled by using Picard tools (v1.34) to remove duplicate reads (optical or PCR), unmapped reads, reads mapping to more than one location, and reads failing vendor QC. Coverage statistics were calculated by using BEDTools (v2.13.2) [21] to assess the quality controlled aligned data. For variant calling, SAMtools (v0.01.18) [22] was initially used to create a ‘pileup’ up of reads in each germline and tumour sample. Somatic variants (SVs) were then identified by using VarScan (v2.3.3) [23] to compare the tumour and normal pileup files and ensure that the absence of germline variation was not due to low sequence coverage. For variant calling using VarScan, the ‘somatic’, ‘somaticFilter’, and ‘processSomatic’ commands were used with default parameters which required ≥6 reads in the germline, a germline variant frequency ≤5%, ≥8 reads in the tumour, ≥3 reads with the variant allele, at least one mutant read on the positive and negative strand, and a tumour variant frequency of ≥10%. Somatic *p*-values were determined by using a Fisher's Exact test to compare variant frequencies in tumour versus germline and a threshold of *p* ≤0.05 was used to select SVs with high confidence.

ANNOVAR [24] was used to annotate the SVs with respect to the gene they occur in, any amino acid changes they cause, and whether they are listed in databases of known variation (dbSNP135, 1000 genomes, Complete Genomics 46, the NHLBI GO Exome Sequencing Project), and an in-house database of 279 non-cancer exomes. This information was used to select novel nonsynonymous SVs for further scrutiny.

To minimise artefactual findings, genes were excluded if they had been identified as possible false positives due to errors in sequence alignment [25] and they were flagged if they had low expression and/or late replication, which are features that have been associated with high rates of somatic mutation [26]. In addition, SVs occurring within or adjacent to repetitive DNA or homopolymers were flagged. As a final check of data quality, the interactive genome viewer [27] was used to inspect the novel SVs and to compare them with germline exomes (n = 61). The novel SVs were also flagged if the same variant was observed at a frequency of ≥10% in any of the germline exomes. All unflagged SVs are shown in Table 3 and counts of flagged and unflagged SVs are shown in Table 2.

The functional significance of variants was determined by using PolyPhen-2 (Polymorphism Phenotyping v2.2.2r398) [28].

To determine whether genes with high confidence SVs showed enrichment of biological function we applied the Gene Functional Classification Tool (http://david.abcc.ncifcrf.gov) to form functionally related groups of genes based on functional data from 14 annotation categories which include Gene Ontology (GO), KEGG Pathways, and OMIM [29].

To investigate copy number changes, VarScan was used to make preliminary copy number calls and circular binary segmentation using the DNAcopy [30] library from BioConductor was used to merge adjacent segments of similar copy number. Segments with log ratios ≥0.7 or ≤-2 were compared across samples to search for regions with recurrent copy number changes.

To assess sample contamination, the non-reference variant calls from SAMtools were used to calculate the ratio of heterozygous to homozygous genotypes in each tumour-normal pair and to estimate the level of contamination by comparison with allele frequency information from the 1000 genomes project [31].

## Results

Firstly, we established that each of the 5 typical HCLc cases evaluated in this study displayed uniform disease subtype features. All cases exhibited mutated *BRAF* V(600)E and imprints of somatic hypermutation (SHM) in *IGHV* genes as determined by standard Sanger sequencing (Table 1). None of the HCLc cases expressed the rearranged Ig *IGHV4-34* gene (Table 1).

Based on the distinctive HCLc immunophenotype, CD19^+^CD11c^+^CD103^+^CD27^-^ tumour cells were purified by flow cytometry and matched CD3^+^CD19^-^ germline T cells flow-sorted from splenocytes, to ~97% and ~98% purity respectively (Fig 1). Genomic DNA was isolated from tumour and germline cells for WES.

Exome library sequencing yielded an average depth of 120-134x with 88–90% of target bases covered by 20 or more reads (Table 4). An additional 63 novel nonsynonymous SVs, with a range of 6–25 variants per tumour, were identified in the 5 HCLc exomes (Table 2). Following comprehensive bioinformatics analysis, >30% of randomly selected putative SVs were re-sequenced by Sanger protocols, and these gave a 100% concordance to SV calls to establish high confidence in the bioinformatic pipeline (data not shown). As expected, the *BRAF* V(600)E mutation was identified in 5/5 HCLc exomes: significantly however, from our data no other recurring mutations were apparent in these HCLc exomes (Table 3).

We evaluated potentially important SVs that occurred only in single individual HCLc genomes in our data set. By structure-function prediction algorithms, the SV in the *DUSP2* (dual specificity phosphatase 2) gene appears to be deleterious (Table 3), and *DUSP2* encodes an inhibitor of ERK in MAPK signalling, to suggest a potential role in potentiating constitutive activation of the MAPK pathway in some HCLc cases. SVs in *CHD7* (chromodomain helicase DNA binding protein 7), *SCD5* (stearoyl-CoA desaturase 5), *SLC22A8* (solute carrier family 22, member 8), and *CLEC6A* (C-type lectin domain family 6, member A) had mutant allele frequencies and corresponding significance levels that were comparable to or more significant than *BRAF* V(600)E (somatic *p*-value ≤1.15x10^-21^, Table 3), pointing to additional drivers that may only occur sporadically in some cases in this form of disease.

The HCLc 5T case has just over twice as many variants as any other sample (22 versus ≤10 SVs) which may be a consequence of a SV in *POLD2* (polymerase [DNA directed], delta 2, accessory subunit) which plays a critical role in DNA replication and repair.

Next we compared our findings of SVs with reported HCL exomes. We identified SVs in *CNTNAP4* (contactin associated protein-like 4), *SLC22A24* (solute carrier family 22, member 24), and *SLC22A8* that are in the same gene family as *CNTN6* (contactin 6) and *SLC5A1* (solute carrier family 5, member 1), which were also reported by Tiacci *et al* in their single HCLc exome study [8]. However, *CNTNAP4* has low expression and late replication that suggests a high rate of somatic mutation and the mutation is predicted to be neutral (Table 3).

Interestingly, very recently data on 3 exomes in *pre-treated* HCLc have been reported, in which mutations in *CDKN1B* were found in 2/3 exomes, in addition to the expected *BRAF* V(600)E [32]. By extending analysis of *CDKN1B* by conventional PCR-based Sanger sequencing means, mutations in this gene were identified in presentation HCLc disease in 10/43 (23%) cases [32]. CDKN1B (p27) is an important cell cycle control protein, and in this extended screen, specific mutations in *CDKN1B* largely differed between individual HCLc patients [32]. In our exome data, we identified a single case (5T) with a *CDKN1B* (C>G p.T198R) mutation (Table 3), differing to the panel of reported *CDKN1B* mutations [32]. Additionally, we identified a mutation in the *ABCC1* gene (ATP-binding cassette, sub-family C member 1) in Case 5T (Table 3), which matches a mutation in the same functional pathway in *ABCA8* gene (ATP-binding cassette, sub-family A member 8) in a pre-treated HCLc case [32], suggesting occurrence prior to therapy and another potential recurrent gene mutation in HCLc disease.

We also observed a further 3 SVs in HCLc that were reported in atypical HCLv-HCLc/*IGHV4-34* exomes [13], in *CREBBP*, *KIAA1755* and *MUC5B* (Table 3). Of these, *CREBBP* and *KIAA1755* have more significant somatic *p*-values in our data, but only the *CREBBP* variant is predicted to be deleterious (Table 3). *CREBBP* encodes a CREB-binding protein, and is one of the earliest epigenetic modifiers that have been described and functions in chromatin remodelling, acetylation and as a scaffold for transcription proteins [33]. *KIAA1755* and *MUC5B* mutations are predicted to be neutral (Table 3). These shared mutations may nevertheless be indicative of shared selective events in specific gene circuits to promote onset of both subtypes of disease.

Comparing our data on exomes in HCLc with the solitary exome reported by Tiacci *et al* [8] and the 3 exomes reported in pre-treated HCLc [32], genes in the solute carrier (*SLC*) and ATP-binding cassette (*ABC*) families may occur more frequently in presentation HCLc disease, and appear to warrant further evaluation by conventional screening in larger cohorts.

## Discussion

Our exome data now establish *BRAF* V(600)E as the *sole* near-universal mutation in presentation HCLc disease, not previously evident from a single exome study [8]. The data, by obviating other highly penetrant mutations, place *BRAF* V(600)E at the centre of the aberrant molecular drive in the pathogenesis and progression in HCLc. It may also explain the exquisite sensitivity of HCLc to Vemurafenib, a targeted inhibitor of mutant BRAF in the clinic [34].

Other recurrent mutations may nevertheless prevail in smaller subsets of HCLc disease. *CDKN1B* gene mutations have now been identified as present in treatment naive HCLc disease in ~23% of cases [32]. Following our comparison of exome mutations in HCL, it is plausible that mutations in the *ABC* and *SLC* gene families may emerge more frequently in cohort screens, to establish specific HCLc subtypes stratified by shared common mutations in addition to *BRAF* V(600)E. However, it appears highly unlikely that they will occur at near-universal levels akin to *BRAF* V(600)E in HCLc, given that 6 exomes have now been evaluated in presentation disease (our data & Tiacci *et al* [8]). In HCLc disease therefore, these data obviate a role for other universal mutation(s) in pathogenesis. Small subsets of HCLc in which other gene mutation(s) are recurrent and are shown to have a driver association may then plausibly combine with mutant *BRAF* to further progress malignancy. Other individual HCLc tumours that display distinct gene mutations that differ entirely between patients may conceptually harbour specific driver mutations that could also co-operate with mutant *BRAF* to promote malignant transformation. The pathways to pathogenesis of HCLc as revealed by exome data consequently appear heterogeneous and varied. It is also noteworthy that in ~50% of HCLv and HCLc/*IGHV4-34* cases, exome data has thus far not identified any common somatic mutations [13], and in these atypical HCL tumours, mutant gene pathways that drive pathogenesis of atypical HCL disease also appear to differ markedly between individual tumours, and are currently not defined.

Our observation that some SVs in HCLc are also found in HCLv-HCLc/*IGHV4-34* exomes [13], in *CREBBP*, *KIAA1755* and *MUC5B*, if verified in larger cohorts of both disease subtypes may reveal shared dysregulated molecular and cellular pathways that are common to origins of HCLc and atypical HCL disease.

Overall, a striking feature is the emergence and relevance of single dominant driver mutations in HCLc and HCLv-HCLc/*IGHV4-34* disease. This is further illuminated when considering the known physiological substrate specificities for BRAF and MEK1 (product of the *MAP2K1* gene). BRAF is a member of the RAF family of cytosolic kinases, and only phosphorylate MEK1/2 [14,15,35]. MEK1/2 kinases in turn mediate phosphorylation that is restricted to ERK1/2, their only two kinase substrates known to date under any physiological condition [14,15]. From these biochemical studies, the substrate specificities intimately link mutant RAF-MEK-ERK drive to the pathogenesis of much of the spectrum of HCL disease. In HCLc, mutant BRAF constitutively activates RAF-MEK-ERK signalling whereas in atypical HCL, it is mutant MEK1 that constitutively activates this pathway. HCL then emerges as an exemplar in cancer of a specific cellular pathway in which genes that are directly functionally linked are mutated in origins of heterogeneous disease subsets.

Examining the RAF-MEK-ERK protein kinases further, these are known to constitute a well-defined and linked signal transduction module or linked enzymatic ‘cassette’ due to the stringent substrate specificities of the RAF and MEK1/2 protein kinases. This linked ‘cassette’ is a global cellular mechanism that can transduce specific receptor-ligand signals initiated at multiple subcellular sites, straddling the outer plasma membrane and different cellular micro-compartments in relaying signals that end in orchestrating specific transcriptional cascades to determine cell fate [36]. An example of receptor-ligand signals transduced by the RAF-MEK-ERK module include BCR-antigen interactions at the cell surface plasma membrane, in which BRAF is the dominant kinase in transducing signals from the BCR to activate ERK1/2 [37]. Another example is the role of this module in mediating regulation of the epidermal growth factor (EGF)-EGF receptor, either at the cell surface plasma membrane or intracellular membranes of the endoplasmic reticulum [36]. A constitutively active RAF-MEK-ERK signalling cassette thereby potentially confers a potent activating mechanism that can drive an array of cellular pathways (reviewed fully in [36]), to progress growth, proliferation and survival in HCL tumour cells. The goal now is to define these activated cellular pathways concisely and examine if they differ between HCLc and atypical HCL disease. Since an identical *BRAF* V(600)E is also found in other diverse lymphoid and solid tumours [16, 38], the activated mutant RAF-MEK-ERK module may by analogy also be exploited by tumour cells to selectively by-pass critical microenvironment receptor-ligand dependencies for survival in those neoplasms.

One of the receptor-ligand interactions relayed by RAF-MEK-ERK signalling is when the BCR is cross-linked by antigen. BCR signals that are transduced by antigen ligation are known to be critical to normal mature B-cell survival [39], but the role of the BCR in malignant HCLc persistence, if any, is as yet not fully defined. HCLc unusually displays multiple pre- and post-isotype switched surface IgM/D/G/A in differing combinations on the same tumour cell [20], and whether each isotype retained functionality in tumour cells had until recently remained unknown. We undertook an evaluation of BCR function in HCLc cells, dissecting the role of individual isotypes, and found unexpectedly that non-IgD isotypes that cross-link the BCR directly trigger apoptosis of tumour cells *ex-vivo* [18]. These data suggested that BCR signals conveyed by non-IgD isotypes are unlikely to support survival of tumour cells. The data also infer that constitutive RAF-MEK-ERK signalling due to *BRAF* V(600)E is unlikely to be required to supplant or by-pass any ligand (antigen) driven BCR-mediated dependency on non-IgD isotypes in HCLc disease. It lends a further complexity as to why multiple isotypes are expressed and retained by individual HCL tumour cells during the course of disease [20].

It is nevertheless now clear from exome data that mutant RAF-MEK-ERK signalling is a central driver mechanism in the pathogenesis and progression of a wide spectrum of HCL disease. Strikingly, the *BRAF* V(600)E mutation has also now been identified in haematopoietic stem cells (HSCs) in HCLc patients [40], revealing acquisition of this signature mutation at a progenitor stage prior to commitment to the B-cell lineage. Despite acquisition of this potent constitutively activated RAF-MEK-ERK pathway in HSCs, current molecular data suggests that malignant transformation occurs in HCLc disease late at the mature B-cell stage, to implicate distinctive pathways of B-cell maturation. Normal B-cells diversify on encounter with antigen when recognised by the BCR to initiate differentiation. In T-cell dependent responses to antigen, B-cells can seed a germinal centre (GC) in which SHM is initiated during a massive blast phase to target *IGV* genes, with the aim of improving fit for antigen, and deletional CSR can follow to alter isotype function [41]. Importantly, human memory B-cells that arise from the GC acquire expression of CD27, a robust marker of GC trafficking [42]. In our previous work, we had delineated that neoplastic arrest in HCLc most likely occurs at a stage of B-cell differentiation that straddles onset of SHM and deletional CSR [20,43], and as HCLc tumour cells do not express CD27 [1], suggests that SHM and CSR may occur at ectopic sites in origins of disease [44]. From this, it appears that antigen driven responses in the periphery may be a vital pre-requisite in *initiating* malignant transformation in HCLc disease, but may not be required in tumour *persistence*, as suggested by our study of BCR isotype function [18].

Significantly, mutant *BRAF* V(600)E has also been identified in HSCs from patients with Langerhans cell histiocytosis (LCH) [45], a condition characterised by pathological accumulation of CD207^+^dendritic cells to indicate that this lesion in HSCs can also drive clonal proliferation in the myeloid cell compartment. Interestingly, onset of LCH has been shown to associate with response to inflammation by dendritic cell progenitors [45], revealing again that normal immune responses are potent driver mechanisms in generating malignant proliferative states from HSC progenitors bearing *BRAF* V(600)E [40,45]. Further stark parallels to HCL disease have also emerged in LCH in relation to the type of somatic mutations found in tumour cells. In LCH disease with wild type *BRAF*, recurrent mutations in the *MAP2K1* gene can be found in up to 50% of cases [46,47], substantiating a role for mutant RAF-MEK-ERK drive, either via *BRAF* V(600)E or mutated *MAP2K1* in LCH pathogenesis.

The findings of *BRAF* V(600)E in HCLc and LCH stem cells also suggests that early selection pressure appears to be operative in funnelling stem cells bearing mutant BRAF to differing cellular lineages. In both malignancies, inflammation-linked responses of lymphoid and myeloid progenitors harbouring mutated BRAF appear to be a critical factor in initiating malignancy, for which there is evidence from knock-in models in LCH [45], but not as yet in HCLc. These pathways also begin to resemble pathogenesis of solid tumours where mutant BRAF in progenitor cells is insufficient to induce full-blown malignancy [16], and additional driver events may well also derive from inflammation-associated interactions with precursor cells, such as those implicated in colorectal cancer [48].

In HCLc specifically then, identifying which additional cellular pathways are aberrantly dysregulated and to what extent, orchestrated by specific genetic mutations and lesions, and how they coalesce with mutant RAF-MEK-ERK drive imparted by *BRAF* V(600)E will unravel a full understanding of malignant origins.
